# TasselNetv2: in-field counting of wheat spikes with context-augmented local regression networks

**DOI:** 10.1186/s13007-019-0537-2

**Published:** 2019-12-11

**Authors:** Haipeng Xiong, Zhiguo Cao, Hao Lu, Simon Madec, Liang Liu, Chunhua Shen

**Affiliations:** 1 National Key Laboratory of Science and Technology on Multi-Spectral Information Processing, School of Artificial Intelligence and Automation, Huazhong University of Science and Technology, Wuhan, 430074 People’s Republic of China; 2 INRA-EMMAH-CAPTE, 84914 Avignon, France; 3 School of Computer Science, The University of Adelaide, Adelaide, SA 5005 Australia

**Keywords:** Wheat spikes, Object counting, Convolutional models, Local regression networks, Context fusion

## Abstract

**Background:**

Grain yield of wheat is greatly associated with the population of wheat spikes, i.e., $spike~number~\text {m}^{-2}$. To obtain this index in a reliable and efficient way, it is necessary to count wheat spikes accurately and automatically. Currently computer vision technologies have shown great potential to automate this task effectively in a low-end manner. In particular, counting wheat spikes is a typical visual counting problem, which is substantially studied under the name of object counting in Computer Vision. TasselNet, which represents one of the state-of-the-art counting approaches, is a convolutional neural network-based local regression model, and currently benchmarks the best record on counting maize tassels. However, when applying TasselNet to wheat spikes, it cannot predict accurate counts when spikes partially present.

**Results:**

In this paper, we make an important observation that the counting performance of local regression networks can be significantly improved via adding visual context to the local patches. Meanwhile, such context can be treated as part of the receptive field without increasing the model capacity. We thus propose a simple yet effective contextual extension of TasselNet—TasselNetv2. If implementing TasselNetv2 in a fully convolutional form, both training and inference can be greatly sped up by reducing redundant computations. In particular, we collected and labeled a large-scale wheat spikes counting (WSC) dataset, with 1764 high-resolution images and 675,322 manually-annotated instances. Extensive experiments show that, TasselNetv2 not only achieves state-of-the-art performance on the WSC dataset ($91.01\%$ counting accuracy) but also is more than an order of magnitude faster than TasselNet (13.82 fps on $912\times 1216$ images). The generality of TasselNetv2 is further demonstrated by advancing the state of the art on both the Maize Tassels Counting and ShanghaiTech Crowd Counting datasets.

**Conclusions:**

This paper describes TasselNetv2 for counting wheat spikes, which simultaneously addresses two important use cases in plant counting: *improving the counting accuracy without increasing model capacity*, and *improving efficiency without sacrificing accuracy*. It is promising to be deployed in a real-time system with high-throughput demand. In particular, TasselNetv2 can achieve sufficiently accurate results when training from scratch with small networks, and adopting larger pre-trained networks can further boost accuracy. In practice, one can trade off the performance and efficiency according to certain application scenarios. Code and models are made available at: https://tinyurl.com/TasselNetv2.

## Background

In agricultural production, crop yield is one of the key factors when monitoring crop growth status. Wheat is one of the top three cereal crops in the world. Its grain yield is mainly associated to $spike~number~\text {m}^{-2}$, $grain~number~\text {m}^{-2}$ and *thousand* *grain* *weight* [1]. Among these traits, $spike~number~\text {m}^{-2}$ is the most important index [2, 3]. Conventional manual approaches to counting wheat spikes are tedious and labor-intensive. The counting results are also error-prone and unrepresentative due to small sampling areas used. To meet the need of large-scale analyses in the era of intelligent agriculture and to obtain the index of $spike~number~m^{-2}$ accurately in real time, counting wheat spikes must be automated in a reliable way, and possibly with low cost.

With the rapid development of recent deep learning technologies, large-scale visual databases and cost-effective graphical processing units, image-based approaches appear to be promising alternatives to automate the task of wheat spikes counting.

**Fig. 1:**
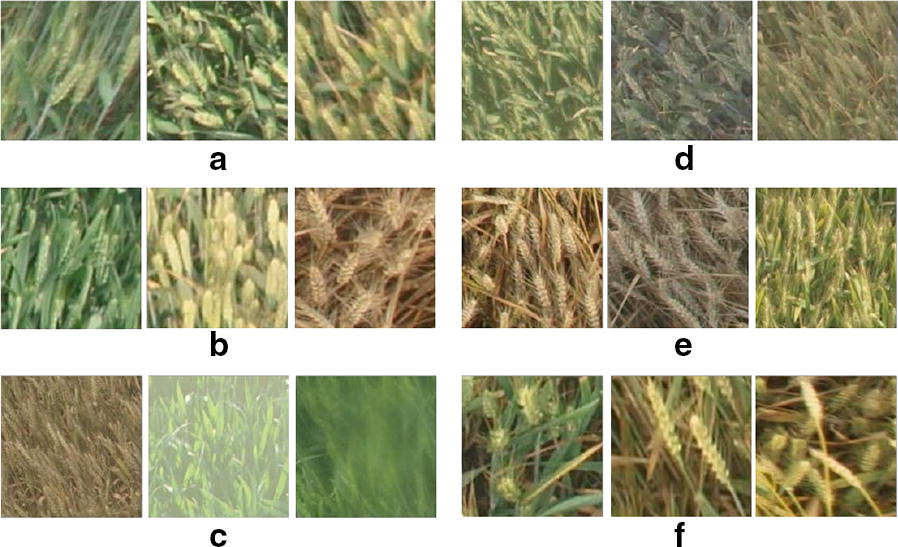
Challenges of counting wheat spikes in the wild. **a** different planting regions, **b** various growth stages, **c** degraded image quality due to blurring, **d** visual differences caused by changing illumination, **e** extremely dense spatial distributions and severe occlusions, **f** size and pose variations

Counting wheat spikes is a typical object counting problem in Computer Vision, and currently convolutional neural network (CNN)-based local regression models have shown remarkable performance in counting crowd [4, 5], vehicles [6], cells [7], animals [8], and plants [9–12]. However, when turning to the scenario of counting wheat spikes in the wild, things are much difficult due to the non-rigid nature of spikes and substantial visual challenges. As shown in Fig. 1, these challenges are:

- Wheats planted in different regions show significant visual differences, due to differences in varieties and geographical environment (Fig. 1a);
- The color, size and shape of wheat spikes vary greatly and unevenly at different growth stages of wheats (Fig. 1b);
- If the imaging equipment lacks manual maintenance, or fog droplets and dust cover the lens, images will be blurred (Fig. 1c);
- Dramatic illumination changes result in completely different visual characteristics of wheat (Fig. 1d);
- The intensive cultivation of wheats gives rise to extremely dense distributions and severe occlusions (Fig. 1e). In these extremely dense areas, even an expert has to count wheat spikes for multiple times to obtain a reliable measure;
- The perspective changes due to the imaging angle. Some wheats may be perpendicular to the lens and only occupy a small number of pixels in the image, which renders difficulties to distinguish wheat spikes from background. This also leads to large size variations of wheat spikes (Fig. 1f).

Above visual challenges make wheat spikes counting a good study case for counting non-rigid objects. Recent literatures emerge on counting wheat spikes but are mainly based on detection. [13–16] first segment the wheats using the RGB images, and then detect each object based on the segmentation result. After detection, the wheat counts can be easily inferred from the objects detected. [17] fuses multi-sensor information (RGB images and multispectral images) to help segmentation. [18] and [19] utilize R-CNN [20] to detect wheat spikes. However, the camera is close to the wheat spikes in these methods, which allows for capturing high-resolution images and obtaining accurate detection but leads to small observation areas. The efficiency of R-CNN processing high-resolution images is also an issue. [21] benefits from active learning to reduce human labeling efforts and use a RetinaNet [22] for detecting and counting sorghum head in UAV-based images in a large region. In order to meet the need of high-throughput plant phenotype analysis over a large area, we leverage images captured from a fixed platform (4 m/5 m above the ground) for counting. These images cover wheat spikes over around 30 $\text {m}^2$. However, wheat spikes present extremely dense distributions and severe overlaps in such images. We notice that non-maximum suppression is regularly used at the end of detection-based methods, which makes it hard to distinguish overlapping objects. Furthermore, there are more than 10,000 wheat spikes in just one image, which makes the bounding boxes annotation nearly impossible. Overall, these counting-by-detection methods render difficulties for counting dense wheat spikes within a large area.

Current state-of-the-art counting approaches typically pursue the idea of local regression with CNNs. Images are often divided into small local patches, and these patches are then processed by the networks individually. Most CNN-based local regression methods adopt density maps as the regression target [4, 5, 23–25]. These methods intend to regress the per-pixel density maps, which is a dense prediction problem. But the problem is that the ground-truth density map is associated with specific choices of Gaussian kernels. This means the ground-truth density map may not be initially accurate, and the error would be introduced before learning the model. To alleviate this problem, [9, 26] prove it is much easier to regress the local count than the density map. The benefit is that the ground truth is no longer sensitive to the exact choice of Gaussian kernels. Lu et al. [9] proposed a local count regression network named TasselNet, which counts maize tassels much more accurate than other existing methods. We believe this idea should also be applicable to other non-rigid objects like wheat spikes.

**Fig. 2:**
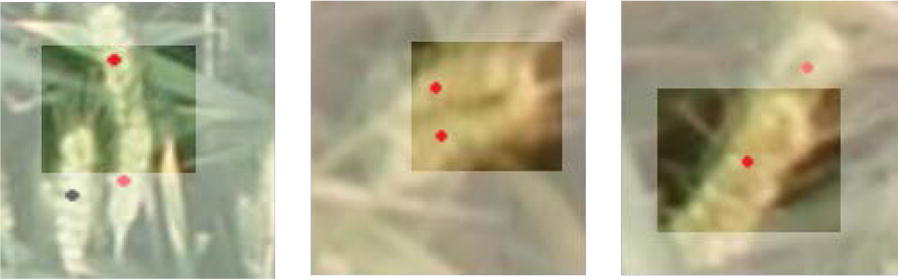
Three examples of incomplete objects when only looking at the local patches. White parts are invisible contextual regions for the current visible patches. Wheat spikes annotated with black dots indicate the spike is partly within the visible area, and red dots represent spikes with severe occlusions. In both cases, accurate wheat numbers are just hard to obtain without the help of local visual context

**Fig. 3:**
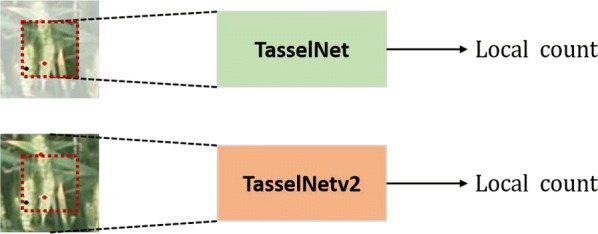
A high-level overview of the approach utilizing local visual context information. The red dashed box indicates a local patch ready for counting, and the part outside the box refers to the context

Albeit successful, we found that TasselNet cannot predict correct counts when spikes partially present in local image patches. As shown in Fig. 2, it is not clear whether there are two wheat spikes or not when only looking at those visible regions. This situation is even more serious when spikes are occluded. In fact, wheats are planted far denser than maize plants, and the density of spikes typically varies between $200/\text {m}^2$ and $600/\text {m}^2$, which means partial spikes would occur frequently in cropped local image patches and thus seriously limits the applicability of TasselNet. To address this, our intuition tells that we need the help of visual contextual information. This is in consistent with the fact that, when one cannot infer the exact number of partially occluded objects within a local area, he may look further until supporting information, such as the border or other object parts, is identified. This kind of supporting information in real world refers to the visual context in images, and it is a kind of “weak context” for it only contains the local surroundings rather than all of remaining images. Therefore, a simple way to tackle above problem is to enable TasselNet to receive both local images and their surrounding pixels, as shown in Fig. 3. This raises a subsequent question: *how to integrate the context into CNNs in a principled way?* One way is to use large convolutional kernels but at the cost of introducing extra parameters. In this paper, we show that a much clever way is to include the context as part of the receptive field so that the model can keep the same number of parameters. This idea is particularly useful for local counting models, such as TasselNet, that do not make full use of their receptive field. As a consequence, we make a simple yet effective extension to TasselNet so that contextual information could be received, leading to an extended version of TasselNet—contextual TasselNet (TasselNetv2 for short).

Another limitation of TasselNet is its low efficiency due to the need of densely sampling local image patches. This introduces many redundant computations. We wonder whether these redundant computations could be avoided in TasselNetv2. Inspired by Fast R-CNN [27], we show that one actually can first extract the features maps of the whole image and then densely sample the feature maps to obtain local features, rather than processing local patches individually. Based on this idea, we implement a fully convolutional form of TasselNetv2, which is proven to be an order of magnitude faster than TasselNet. In particular, we created a large-scale Wheat Spikes Counting (WSC) dataset to validate the effectiveness of TasselNetv2.

Extensive experiments show that, TasselNetv2 reaches $91.01\%$ relative counting accuracy and achieves the state-of-the-art performance on the WSC dataset, and notably, can process images 13.21 times faster than TasselNet (13.82 fps for TasselNetv2 vs. 1.05 fps for TasselNet). Further experiments demonstrate that TasselNetv2 also reports state-of-the-art counting performance on the Maize Tassels Counting (MTC) and ShanghaiTech Crowd Counting datasets [5], which confirms a good generality of TasselNetv2. Several interesting ablative studies are conducted to justify the effectiveness and necessity to include the context for better counting performance.

Overall, the main contributions of this paper are:

- We introduce a principled way to supplement the local visual context into convolutional models by treating it as part of the receptive field, which can improve the counting performance without increasing extra parameters;
- We propose a simple yet effective extension of TasselNet to its contextual version TasselNetv2. TasselNetv2 not only improves the counting performance but also speeds up the computation with an order of magnitude;
- We collect and annotate a large-scale WSC dataset with 1764 high-resolution images and 675,322 manually-labeled instances;
- We report state-of-the-art counting performance on the WSC, MTC and ShanghaiTech datasets.

## Method

### Image acquisition

Field wheat images in the WSC dataset are collected from three experimental fields of Gucheng, Hebei, Zhengzhou, Henan, and Tai’an, Shandong, containing seven sequences from 2011 to 2013. Due to the different local geology and climate conditions, three cultivars were planted, respectively, including Zimai No. 24 in Taian, Jimai No. 22 in Gucheng, and Zhengmai No. 366 in Zhengzhou.

**Fig. 4:**
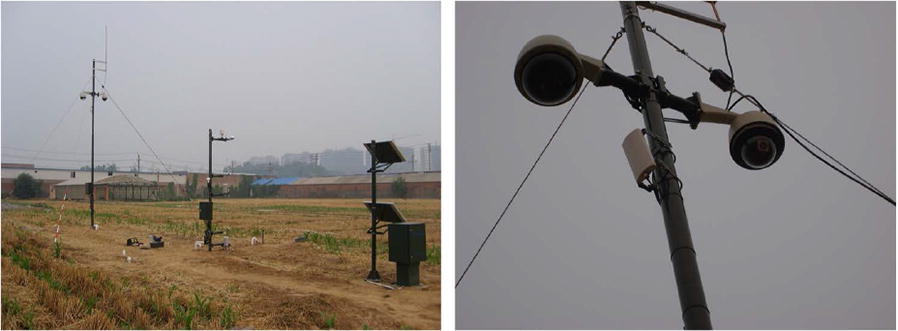
Imaging device in the Zhengzhou, Henan Province. The main components include a high resolution CCD digital camera (E450 Olympus) and low-resolution monitoring equipment. The camera is set 5 m high above the ground

Figure 4 shows the image capturing device, main components include a high-resolution CCD digital camera (E450 Olympus), a low-resolution monitoring equipment, a 3G wireless data transmission system, and several solar panels for power supply. The CCD digital camera is set with a height of 5 m above the ground, and the focal length is fixed to 16 mm. From 8 a.m. to 17 p.m., images are captured from a perspective oblique to the ground once an hour. After images are acquired, wheat images are transmitted to the remote server through the 3G wireless network, and then we can access the image data. For detailed information of the image capturing equipment, readers can refer to [28].

### Wheat spikes counting dataset

There are tens of thousands of wheat spikes in the wheat images, and they present a high degree of similarity when the time interval is short, which makes the annotations for all of the captured images costly and needless. This means only a subset of images is essential to build the dataset, but this subset should be large enough to cover wheat spikes in various scenarios. We pick out this subset with a two-stage selection strategy. At the first stage, we choose images according to the date, after the heading stage of wheat. Before obvious emergence of spikes, the sampling interval is set to 3 days. After wheat spikes emerge, the number of wheat spikes changes rapidly, and thus the sampling interval is shortened to 2 days. At the second stage, 10 candidate images collected in each day (from 8 a.m. to 17 p.m.) are taken into account. Considering the illumination characteristics in one day, three images are chosen from three time periods, i.e., morning (8 a.m. to 11 a.m.), noon (12 a.m. to 14p.m.), and afternoon (15 p.m. to 17 p.m.), to maintain the diversity of the dataset.

Finally, a total of 196 images, with the resolution of $3648\times 2736$, were chosen. The number of wheat spikes varies from 0 to over 10, 000. Since the image resolution is very high, and wheat spikes are extremely dense (it brings tremendous difficulties for the annotation process), each original image is cropped to 9 sub-images with a resolution of $1216\times 912$. Thus, 1764 images in all are used to construct the dataset. Table 1 presents the information of each sequence in the dataset.

**Table 1:** Constitution of the WSC dataset

Sequence	Images	Spikes	Min	Max
Hebei Gucheng (2011–2012)	324	82,578	0	661
Henan Zhengzhou (2011–2012)	234	118,022	0	1462
Henan Zhengzhou (2012–2013)	171	104,847	0	1331
Shandong Taian (2011–2012 Camera 1)	279	97,695	0	1010
Shandong Taian (2011–2012 Camera 2)	261	78,887	0	908
Shandong Taian (2012–2013 Camera 1)	234	94,454	0	1090
Shandong Taian (2012–2013 Camera 2)	261	98,839	0	971
Total	1764	675,322	0	1462

*Images* denote the number of images in each sequence. *Spikes* refer to the number of wheat spikes in each sequence. *Min* and *Max* indicate the minimum and maximum number of wheat spikes per image

With seven sequences in the WSC dataset, the training set, validation set and test set are divided, as shown in Table 2. Images from the Shandong Taian (2012–2013 Camera 1) sequence exhibit a relatively clear distinction between spikes and background. Spikes in this sequence also appear to have a high density and are with dramatic changes caused by illumination. In the Henan Zhengzhou (2012–2013) sequence, it is hard to distinguish the spikes from the background. The presence of severe occlusions makes this task even more challenging. Evaluations on these sequences can sufficiently show the adaptability and robustness of the counting method. Local visual context may be helpful for identifying overlapped objects, as shown in Fig. 2. We embed local visual context in TasselNetv2 to alleviate such a problem.

**Table 2:** Training set (train), validation set (val) and test set (test) settings of the WSC dataset

Sequence	Train	Val	Test
Hebei Gucheng (2011–2012)	$\checkmark$	$\checkmark$	
Henan Zhengzhou (2011–2012)	$\checkmark$	$\checkmark$	
Henan Zhengzhou (2012–2013)			$\checkmark$
Shandong Taian (2011–2012 Camera 1)	$\checkmark$	$\checkmark$	
Shandong Taian (2011–2012 Camera 2)	$\checkmark$	$\checkmark$	
Shandong Taian (2012–2013 Camera 1)			$\checkmark$
Shandong Taian (2012–2013 Camera 2)	$\checkmark$	$\checkmark$	

**Fig. 5:**
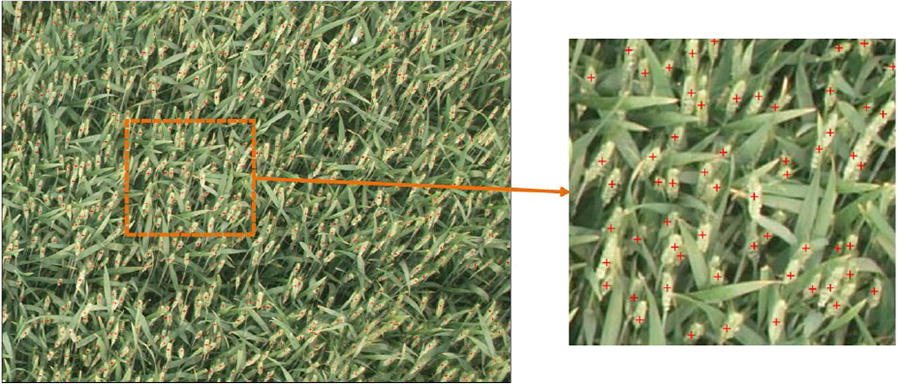
An example of dotted annotation. A red dot is marked at each location of the wheat spike

Following [9], dotted annotation is adopted where a point is marked at the location of each wheat spike. Figure 5 shows an example of annotated image. Six colleagues in our laboratory first participated in the annotation process. After the dataset is annotated, we double-checked the annotations and corrected some missing and wrong annotations. Especially for the second round checking, we trained a TasselNet to predict counts and identified the areas with high counting errors. With this kind of auxiliary information, particular attentions are paid to these areas for careful checking further, and other areas are also checked again.

### Design of TasselNetv2

We first highlight the concepts of “input image”, “input patch” and “input patch with context” in Fig. 7. They are prerequisites for readers to better understand TasselNetv2.

Local patches from an image may have severe overlaps due to dense sampling, but TasselNet requires extracting the local feature from each patch first and then mapping it to the local count. In this paradigm, many redundant calculations appear during feature extraction. Inspired by Fast R-CNN [30], redundant calculations can be avoided by first extracting the feature maps of the whole image, then densely sampling the feature maps to obtain local features and finally mapping them to local counts in a light-weight manner.

Notice that fully-connected layers in TasselNet can also be implemented as convolutional layers with $1\times 1$ kernels [31]. When the convolutional kernel slides over the image and manipulates a local area of pixels at a time, it performs a form of dense sampling. This inspires us to replace the explicit dense sampling with convolution.

#### Motivation

The local visual context, in the framework of local regression, refers to the surrounding pixels of local sampling patches. In Fig. 2, if the visible parts belong to local sampling patches, those invisible parts represent the context. Unfortunately, since the context is not within local patches, it remains invisible to local regression networks like TasselNet. If a network can see the context, overlapping objects or part of objects may be inferred easily and counted accurately. The high-level idea is thus to enable the network to process both local patches along with the context, as shown in Fig. 3.

#### Adding context

**Fig. 6:**
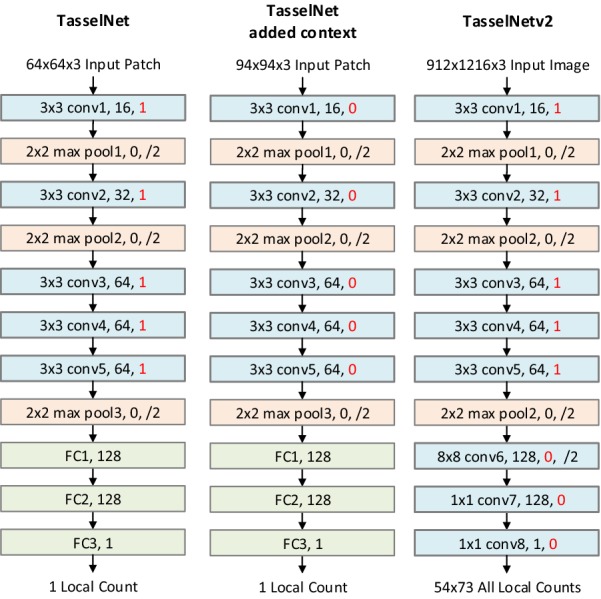
The structure of TasselNet, TasselNet added context and TasselNetv2. All of the networks adopt AlexNet-like architectures. The definition of the convolutional and pooling layers is in the format: fliter size + layer name, number of channels, padding, /stride. Fully connected layers are defined in the format: layer name, number of nodes. The different settings are highlighted in red

The main idea of TasselNetv2 is to process local patches with the context. Notice that there is a massive waste of the receptive field in TasselNet. It is natural to think how to reduce such a waste. In this paper, we show that one can cancel zero paddings to enable the network receiving extra context and to make full use of the receptive field. The way to achieve this is simply to delete paddings in all of convolutional layers, as shown in Fig. 6.

**Fig. 7:**
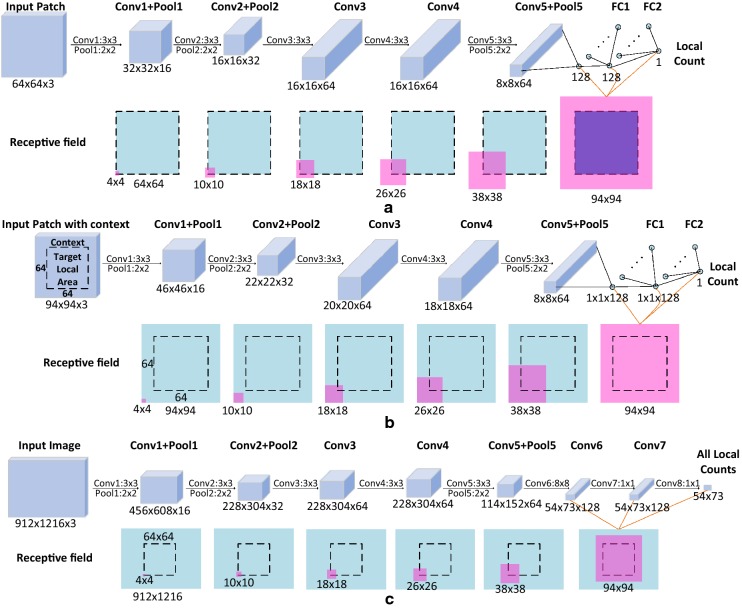
Feature maps and the corresponding receptive field of TasselNet and TasselNetv2. **a** For TasselNet, **b** for adding context to TasselNet via canceling zero-paddings and **c** for TasselNetv2. The above line are feature maps of each layer in the network, numbers below feature maps are in the format: $height\times width\times channel~numbers$. The following line is the corresponding receptive fields, where black dotted boxes represents the target local area to be counted, the blue rectangular areas represents the input area, and the pink area represents the receptive field of the bottom left element in the feature map (the part of the receptive field beyond the input area denotes zero area). Since the last few layers have receptive fields of the same size, we use orange lines to point to the corresponding receptive fields

We explain why this simple modification makes sense through a visualizing analysis of the receptive field in Fig. 7, and a brief introduction about computing the receptive field is also provided in Additional file 1. Assume TasselNet and TasselNetv2 regress the local count of the $64\times 64$ local area. TasselNet (a) receives the local area without the context. It has zero paddings in all convolutional layers, and these paddings cause the zero area in the receptive field outside borders. However, if removing all the zero paddings, TasselNet (b) can leverage the wasted receptive field to receive extra context and keep the same amount of parameters.

It is worth noting that, though the network processes $94\times 94$ patches, it still regresses local counts aggregated from the central $64\times 64$ areas. Many counting approaches assume that CNNs are able to identify each object within their local receptive fields [26, 29], while we argue that *one should treat part of the local receptive field as additional context* towards accurate counting. This is what makes TasselNetv2 quite different from existing CNN-based local regression models.

#### Improving efficiency

Inspired by the idea of fully convolutional networks (FCNs) [32], we implement TasselNetv2 into a fully convolutional form, which speeds up both training and inference significantly, as shown in Fig. 6. In what follows, we further explain in detail how TasselNetv2 works and improves efficiency.

TasselNetv2 is a composition of convolutional layers. If skipping the activation functions, the composition of convolutional layers can be view as a convolutional layer with a large kernel, and the filter size equals to the size of the receptive field. As shown in Fig. 7, the size of the receptive field of the output remains $94\times 94$, so TasselNetv2 can be seen as a large $94\times 94$ convolutional layer and maps each $94\times 94$ local area (local patch with context) to a local count. Meanwhile, since four layers are with a stride of 2, this large convolutional filter slides with a stride of $2^4=16$, which is equivalent to densely sampling the input image with a stride of 16. As a consequence, TasselNetv2 adds context into TasselNet in a FCN-like manner. It is worth noting that the context is naturally exploited in FCNs by most local areas. Only the context close to image borders is partially utilized by TasselNetv2, e.g., the local area in the upper left corner only has the lower right part of the context. In order to keep the size of these local areas to be $94\times 94$, we supplement 15 zero paddings around the image borders. An elegant way to embed this pre-processing in TasselNetv2 is to use the accumulation of zero paddings from the first five layers (these zero paddings accumulate to 15 zero paddings around the input image).

The calculations performed in CNNs are mainly Floating Point Operations (FLOPs), and FLOPs are also widely adopted in evaluating the computation complexity of CNNs [33, 34] from the view of computation amount. We remark the efficiency of TasselNetv2 using FLOPs during testing in Table 3. The first five convolution layers extract feature maps, and the following three layers map features to local counts. As mentioned in [9], dense sampling is essential to generate adequate training samples for TasselNet. However, $10\times$ extra calculations are needed in this paradigm, compared to sampling non-overlapping patches. This is due to the redundant computations in both feature extraction and feature mapping. Instead, TasselNetv2 directly extracts the feature maps of the whole image, densely samples local features from the feature map and maps them to local counts simultaneously. In this way, TasselNetv2 avoids redundant calculations during feature extraction and is thus much more efficient than TasselNet. It can directly process the whole image and regress all local counts with a single forward pass.

**Table 3:** Comparison towards the floating point computations (FLOPs) when processing images with the resolution of $1216\times 912$. Only the single-precision floating point multiplication are taken into account

	TasselNet		TasselNetv2
	Non-overlap	Dense sample	
conv1	$4.70\times 10^{8}$	$6.92\times 10^{9}$	$4.79\times 10^{8}$
conv2	$1.24\times 10^{9}$	$1.83\times 10^{10}$	$1.28\times 10^{9}$
conv3	$1.22\times 10^{9}$	$1.81\times 10^{10}$	$1.28\times 10^{9}$
conv4	$2.44\times 10^{9}$	$3.61\times 10^{10}$	$2.56\times 10^{9}$
conv5	$2.44\times 10^{9}$	$3.61\times 10^{10}$	$2.56\times 10^{9}$
conv6(fc1)	$5.17\times 10^{8}$	$2.07\times 10^{9}$	$2.07\times 10^{9}$
conv7(fc2)	$1.75\times 10^{7}$	$6.46\times 10^{7}$	$6.46\times 10^{7}$
conv8(fc3)	$1.26\times 10^{5}$	$5.05\times 10^{5}$	$5.05\times 10^{5}$
Total	$8.34\times 10^{9}$	$1.16\times 10^{11}$	$1.03\times 10^{10}$

### Inference of TasselNetv2

**Fig. 8:**
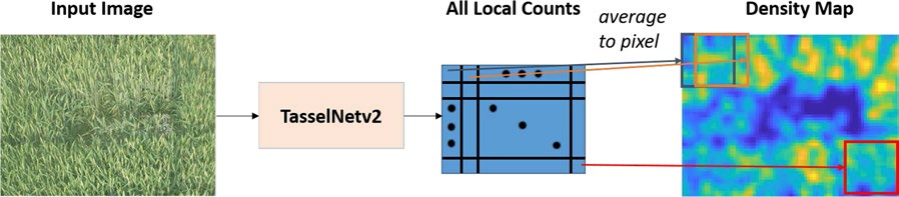
The processing pipeline of TasselNetv2 at the test stage. Unlike TasselNet, TasselNetv2 directly processes the whole input image and outputs all local counts. And the final density map can be acquired by merging and normalizing all local counts

Here we formally introduce the processing pipeline of TasselNetv2 during inference, as shown in Fig. 8. TasselNetv2 directly processes the whole image of arbitrary size (in this paper, the whole image refers to the image of size $1216\times 912$) and regresses all local counts at the same time. However, since individual local areas have overlaps, the global image count cannot be acquired by summing over the whole count map directly. Following the aggregation and normalization strategy mentioned in [9], all local counts are merged to obtain the normalized count map. After normalization, the global image count can then be reflected by integrating over the count map.

### Implementation details

We implement TasselNetv2 based on MatConvNet [35]. During training, we use 1359 images in the training and validation sequences of the WSC dataset. $90\%$ images are randomly chosen for training, while the rest for validation. Before learning, mean subtraction is preprocessed (the mean is computed from the training set). It is worth mentioning that, no data augmentation is performed because the WSC dataset already contains wheat spikes under various scenarios.

We initialize networks with the *improved Xavier* method [36]. The standard stochastic gradient descent is applied to optimize the parameters of the network. The learning rate is initially set to 0.1 and is decreased when the training error stagnates. To speed up and stabilize the error convergence process, a batch normalization layer [37] is attached after each convolutional layer before ReLU.

The training time of TasselNetv2 on the WSC dataset varies from 4 h to 2 days depending on the network architecture used (4 hours for the Alex-like architecture, and 2 days when the pretrained VGG–16 is used). When training TasselNet on the WSC dataset, the training time varies between 4 days and 2 weeks according to the network capacity used (Matlab 2017a, OS: Window10 Home 64-bit, CPU: Intel i7-7700 3.60GHz, GPU: Nvidia GeForce GTX 1070 (8GB), RAM: 16 GB).

## Results and discussion

Extensive experiments are conducted to demonstrate the effectiveness and efficiency of TasselNetv2. First, we perform experiments on the WSC dataset to search optimal hyper parameters. After obtaining these, we verify the effect of adding context in TasselNetv2. Next, TasselNetv2 is further compared against other state-of-the-art approaches on the WSC dataset. To demonstrate the generality of TasselNetv2, we also evaluate it on the MTC [9] and ShanghaiTech datasets [5].

Mean absolute error (*MAE*) and root mean squared error (*RMSE*) are chosen to quantify the counting performance. They are defined as

1 $$\begin{aligned} \small MAE=\frac{1}{N} \sum _{i=1}^{N} \left|C^{pre}_{i}-C^{gt}_{i}\right|\,, \end{aligned}$$

2 $$\begin{aligned} \small RMSE=\sqrt{ \frac{1}{N} \sum _{i=1}^{N} \left(C^{pre}_{i}-C^{gt}_{i}\right)^{2}}\,, \end{aligned}$$

where *N* denotes the number of images, $C^{pre}_{i}$ denotes the predicted count of the *i*-th image, and $C^{gt}_{i}$ denotes the corresponding ground-truth count. *MAE* measures the accuracy of counting, and *RMSE* measures the stability. Lower *MAE* and *RMSE* imply better counting performance.

### Searching optimal parameters

Since TasselNet is the direct baseline of TasselNetv2, we set the hyper parameters of TasselNetv2 same as the TasselNet, in order to demonstrate the superiority of TasselNetv2 w.r.t. TasselNet and the benefit of embedding context information. Hence, we first search the optimal parameters on the WSC dataset using TasselNet so that TasselNet can report the optimal performance, and we then apply the same parameters to TasselNetv2.

Through extensive experiments, the optimal setting of hyper parameters for TasselNet on the WSC dataset is summarized in Table 4. Detailed procedures of searching optimal parameters are provided in Additional file 1.

**Table 4:** TasselNet configurations on the WSC dataset

Patch size	$64\times 64$	Gaussian size	4
Backbone of TasselNet		AlexNet-like in Fig. 6	

### Why adding context?

#### Adding context is effective

We first compare TasselNet trained with/without the context to highlight the pure effect of adding the context. Then, TasselNetv2 is evaluated to show its efficiency and accuracy beyond TasselNet.

Quantitative results are presented in Table 5. We observe that, when forcibly adding the context into TasselNet during only inference (trained without context), the counting error increases notably, which suggests that TasselNet cannot utilize contextual information when trained without the context. This is the problem we call *information asymmetry*. However, after embedding contextual information since the training phase, the MAE decreases more than 10 without increasing model parameters (compared to TasselNet). Adding the context is effective. It is worth noting that this significant performance improvement comes almost at no cost.

**Table 5:** The effect of context on the test set of the WSC dataset. “train” denotes adding context into TasselNet since training phase as Fig. 7b, while “test” denotes only adding context into TasselNet in the testing phase

Method	Context	MAE	RMSE	Train (s)
TasselNet	$\times$	61.35	99.27	3495.29
TasselNet	Test	79.42	126.18	3495.29
TasselNet	Train	*50.17*	82.16	4026.68
TasselNetv2	$\checkmark$	50.79	*80.66*	333.27

All networks are trained from scratch. Training time for one epoch is reported. The best performance is in italics

It also can be observed that TasselNetv2 exhibits the same degree of improvement of adding the context. Meanwhile, TasselNetv2 is more than 10 times faster than TasselNet during the training stage. This is achieved by processing input images in a FCN manner rather than densely sampling image patches, thus avoiding redundant computations in feature extraction, as analysed in Table 3. Now we can say that TasselNetv2 is a much more efficient implementation of adding the context into TasselNet.

**Fig. 9:**
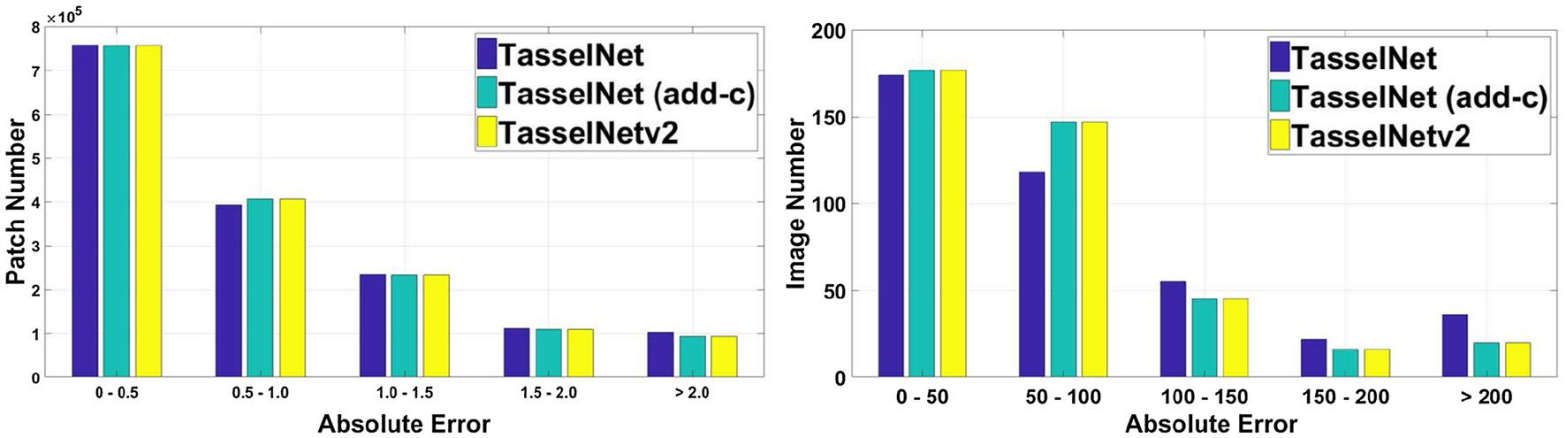
The distribution of absolute errors for local patches and test images. The left is the histogram of absolute error for local patches, and the right is the histogram of absolute error for test images. All networks are trained from scratch. “TasselNet (add-c)” denotes adding the context in TasselNet as per Fig. 6 since the training phase

We further analyze the error distributions in Fig. 9, and find that patch-based and image-based errors are more likely to shift towards zero with the help of context. So far, it can be concluded that lacking the context is the main drawback of TasselNet, and it is important to add the context during training.

#### Adding context is necessary

Notice that we treat the context as part of the receptive field and regress only the local count from the central region. One may wonder what if the network simply regresses the local count accumulated from the whole receptive field. Another baseline *TasselNetv2 (del-c)* is used to justify this point, where we delete the context of the input patch in TasselNetv2. Specifically, we alter the regression target of TasselNetv2 to the object count within the whole $94\times 94$ receptive field (rather than the $64\times 64$ central area in our proposition).

According to the results in Table 6, we can see that the counting performance of TasselNetv2 (del-c) drops significantly (66.96 MAE), even worse than TasselNet. This implies a network may not sense everything in its receptive field. A possible explanation may be given from some recent findings on the effective receptive field. First, the effective receptive field is much smaller than the theoretical receptive field [38]. According to [39], the effective receptive field empirically obeys a Gaussian distribution, which means pixels close to the center of the receptive field have much larger impact on counting than marginal pixels close to the boundary of the receptive field. A network may not capture sufficient evidence to support regressing counts at the border of the receptive field, while our empirical study shows that adding the context into part of the receptive field as auxiliary information can help to improve the counting of objects located in the center of receptive field.

**Table 6:** The necessity of adding context on the test set of the WSC dataset

Method	MAE	RMSE
TasselNet	61.35	99.27
TasselNetv2	*50.79*	*80.66*
TasselNetv2(del-c)	66.96	113.20

All networks are trained from scratch and with the same hyper parameters. The best performance is in italics

The above experiments justify that it is better to use a portion of the receptive field as the context, instead of counting all objects within the whole receptive field [26].

### Comparison with state of the art

According to the above evaluations, the optimal setting on the WSC dataset is shown in Table 4. Next, to compare TasselNetv2 with other state-of-the-art methods, several well-established baselines are chosen:

- Segmentation method in [13]: This is the latest counting by segmentation method specially designed to count wheat spikes in the field. It first applies Laplacian frequency filtering to remove background, then utilizes the median filter to eliminate noise, and finally, finds the maximal to split individual wheat spikes;
- Density map regression methods: CCNN [6] and MCNN [5] are two typical counting-by-regression methods, which aim to regress pixel-wise density maps. Their parameters are of the same order of magnitude as TasselNetv2. CSRNet [23] represents the state-of-the-art crowd counting approach and is composed of a much deeper CNN (pretrained VGG16) as the front-end used for feature extraction. For a fair comparison, we replace the feature extractor in TasselNetv2 (the first 5 convolutional layers) with all convolutional layers in VGG16 [40] and mark it as TasselNetv2$^\dagger$. More details about TasselNetv2$^\dagger$ can be found in Additional file 1.
- Local count regression method: TasselNet [9] regresses the local counts rather than density maps. This is our direct baseline and the most closely-related approach. A brief introduction to TasselNet can also be found in Additional file.

Results are listed in Table 7. We can make the following observations:

- Segmentation method in [13] works poorly on the WSC dataset (317.19 MAE). Due to heavy dependency on the color information, this method is very sensitive to the illumination that significantly changes the color attributes. This also implies the problem of counting wheat spikes in the field-based environment cannot be addressed just by segmentation.
- Density map regression methods, such as CCNN and MCNN, perform much better than the segmentation method, with 101.39 MAE and 97.08 MAE, respectively. It seems that these two CNN-based methods can adapt to the in-field environmental variations and the morphological variations of wheat spikes to a certain degree. Nevertheless we remark that density map prediction may not be suitable for counting wheat spikes, because the ground-truth density map cannot be generated accurately. This is also true for counting other non-rigid objects.
- TasselNet outperforms CCNN and MCNN on the WSC dataset (61.35 MAE). It considerably reveals the benefit of local counts regression, which is important for object counting problems that have size variations.
- CSRNet slightly outperforms TasselNetv2 (46.32 MAE versus 50.79 MAE). However, CSRNet not only has substantial parameters, more than an order of magnitude compared to TasselNetv2, but also is greatly benefited from the pre-trained model. Though with these unfair factors, TasselNetv2 still exhibits comparable performances against CSRNet. When TasselNetv2$^\dagger$ uses the same pretrained VGG16, it outperforms CSRNet, with 44.27 MAE ($91.01\%$ relative counting accuracy), reaching the state-of-the-art performance on the WSC dataset. As a consequence, for time-sensitive applications, TasselNetv2 is still our recommended choice.

**Table 7:** Comparison with state-of-the-art counting approaches on the test set of WSC dataset. TasselNetv2 adopts an AlexNet-like architecture in Fig. 6 and is trained from scratch

Method	Henan Zhengzhou (2012–2013)		Shandong Taian (2012–2013 Camera1)		Overall		#Parameters
	MAE	RMSE	MAE	RMSE	MAE	RMSE	
Segmentation method in [13]	387.09	436.84	268.03	345.78	317.19	386.22	$\times$
CCNN [6]	168.41	214.41	52.40	72.78	101.39	149.91	$5.70\times 10^5$
MCNN [5]	149.44	188.34	58.83	75.50	97.08	135.17	$1.33\times 10^5$
CSRNet$^\dagger$ [23]	64.19	88.96	33.26	*46.19*	46.32	67.63	$1.63\times 10^7$
TasselNet [9]	94.97	137.24	36.79	57.37	61.35	99.27	$6.38\times 10^5$
TasselNetv2	74.97	113.21	33.12	49.26	50.79	80.66	$6.38\times 10^5$
TasselNetv2$^\dagger$	*61.57*	*87.67*	*31.62*	47.55	*44.27*	*67.47*	$1.60\times 10^7$

$^\dagger$ means the model is finetuned from the pretrained VGG16, and layer-by-layer settings can be found in Additional file. The best performance is italics

### Evaluation on the MTC dataset

To show that TasselNetv2 is a generic object counting method, particularly for the application in the agriculture scenario. We further evaluate the effectiveness of TasselNetv2 on the Maize Tassels Counting (MTC) [9] dataset, following the same setting as [9]. Detailed results are shown in Table 8.

**Table 8:** Evaluations of different methods on the MTC [9] dataset

Method	MAE	RMSE
JointSeg [41]	24.2	31.6
mTASSEL [42]	19.6	26.1
GlobalReg [43]	19.7	23.3
DensityReg [44]	11.9	14.8
CCNN [6]	21.0	25.5
TasselNet [9]	6.6	9.6
TasselNetv2	5.4	*8.8*
TasselNetv2$^\dagger$	*5.3*	9.4

$^\dagger$ means the model is finetuned from the pretrained VGG16. The best performance is in italics

TasselNet currently represents the state-of-the-art approach on the MTC dataset. According to the results, we found that TasselNetv2 outperforms TasselNet and further reduces the counting error by $18.2\%$ (5.4 MAE versus 6.6 MAE). The context is also an important factor for maize tassels.

With a pre-trained model, TasselNetv2$^\dagger$ only performs slightly better than TasselNetv2 but increases more than an order of magnitude of parameters. We conjecture the main reason is the lack of training samples in the MTC dataset (only 186 training images). The potential of pre-trained models may not be fully exploited with such a small dataset, while a small network, such as TasselNetv2, can already produce satisfactory results. In this case, TasselNetv2 is effective and efficient, which seems to be a better choice than TasselNetv2$^\dagger$.

### Evaluation on the ShanghaiTech dataset

We further evaluate TasselNetv2 on the ShanghaiTech dataset [5] to see its generality to crowd counting, following the same experimental setting in [5]. Results are listed in Table 9.

**Table 9:** Evaluations on the ShanghaiTech [5] dataset

Method	Part A		Part B	
	MAE	RMSE	MAE	RMSE
MCNN [5]	110.2	173.2	26.4	41.3
CP-CNN [25]	73.6	106.4	20.1	30.1
ACSCP [24]	75.7	*102.7*	17.2	27.4
CSRNet$^\dagger$ [23]	68.2	115.0	10.6	*16.0*
TasselNet [9]	87.0	138.9	16.7	28.1
TasselNetv2	84.1	140.1	15.3	27.8
TasselNetv2$^\dagger$	*66.8*	112.1	*9.6*	17.5

$^\dagger$ means the model is fine-tuned from the pretrained VGG16. The best performance is in italics

On both the part A and part B subsets, the benefit of adding the context can be reflected when comparing TasselNetv2 with TasselNet, but the improvement is marginal. When using a pre-trained VGG-16 model, TasselNetv2$^\dagger$ outperforms CSRNet and reaches the state-of-the-art performance. This suggests pre-trained models is necessary to fully exploit the benefit of context on the ShanghaiTech dataset.

### Some failure cases

**Fig. 10:**
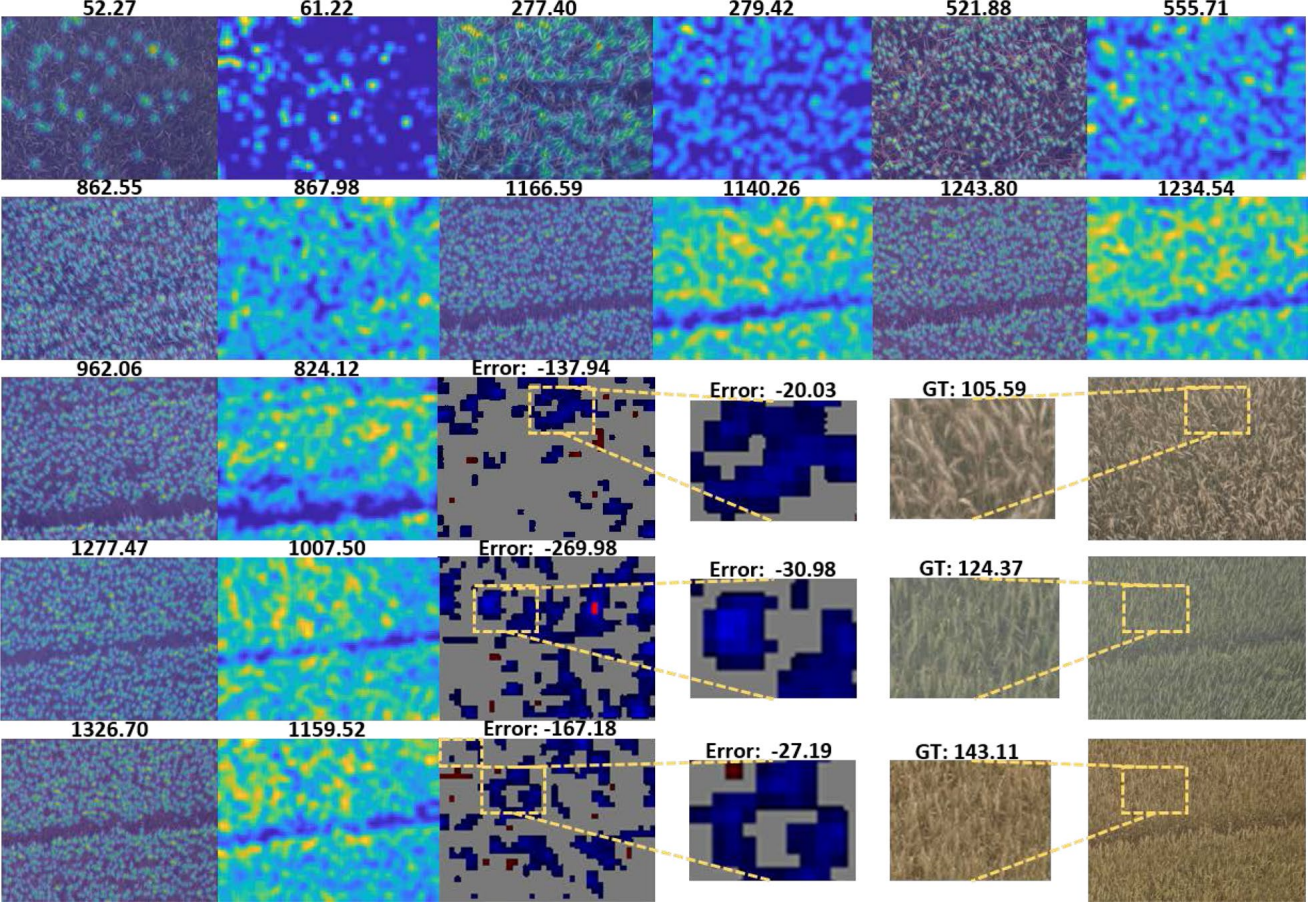
Some ground truth density maps overlaid on original images on the test set of the WSC dataset and count maps generated by TasselNetv2 (finetuned with pre-trained VGG16). The number above each original image denotes the ground truth count number of wheat spikes, while that above each density map denotes prediction count number. The last line shows some unsuccessful predictions, and error maps of these images are also presented. An error map denotes the difference of the ground truth and predicted density map. Over-estimate is denoted by red, under-estimate by blue, and minor difference by gray. The darker the color is, the greater the errors are. We also zoom in some local areas with high counting errors. ’GT’ denotes ground-truth counts and ’Error’ denotes the difference compared to the ground truth. Further visualizations can be found in Additional file 1.

Figure 10 shows some qualitative results of TasselNetv2 on the WSC dataset. In most cases, TasselNetv2 predicts accurate counts (the first four rows). However, it exposes prominent under-estimate phenomena in some cases, particularly when severe overlapping and heavy blurring occur. These visual patterns raise a huge challenge to discriminate spikes even for a human expert. Efforts still should be paid to overcome these challenges. We leave this for future explorations.

## Conclusions

In this work, we addressed an important and practical problem of counting wheat spikes in the field-based environment using computer vision. We observe that, some existing CNN-based local regression models, such as TasselNet, suffer from the problem of lacking contextual information, so they usually cannot predict correct counts when objects partially present in local image patches. By integrating the context into the framework of the TasselNet, we proposed a simple but effective extension, i.e., TasselNetv2. A large-scale WSC dataset, with 1, 764 images and 675, 322 annotated wheat spikes, is also created. The dataset is very challenging due to intrinsic and extrinsic variations not only in spikes per se but also in environment, which makes it appropriate to be used as a benchmark for counting non-rigid objects.

Extensive experiments illustrate that, TasselNetv2 achieves state-of-the-art performance on the WSC dataset with $91.01\%$ relative counting accuracy, and is also more than an order of magnitude faster than TasselNet. Further evaluations on the MTC and ShanghaiTech datasets demonstrate that TasselNetv2 can also push forward the state of the art. Sufficient analyses of potential issues effecting the practical application of TasselNetv2 are also described, including emphasizing the role of the context in object counting, searching optimal parameters for local counts regression, and analyzing potential errors. We believe TasselNetv2 shows great potentials to be applied to other object counting domains.

Albeit empirically effective, the reason why the context can improve the counting performance only stays at an intuitive level, and it remains unclear how the context interacts with the central receptive field as auxiliary information. We hope such empirical findings in this paper could inspire others to uncover the mystery of the receptive field.

## Supplementary information

**Additional file 1.** More details about the WSC dataset, experiment settings and results. A brief introduction and analysis to the TasselNet [9] are also included.

## Data Availability

The WSC dataset and other supporting materials are made available online at: https://tinyurl.com/TasselNetv2.
