# Supplementary material for: TasselNetv2: in-field counting of wheat spikes with context-augmented local regression networks
Source: Plant Methods. 2019 Dec 11;15:150. doi: 10.1186/s13007-019-0537-2 (PMC6905110; doi:10.1186/s13007-019-0537-2)
Supplement: Supplementary file 1 — Additional file 1. More details about the WSC dataset, experiment settings and results. A brief introduction and analysis to the TasselNet [9] are also included. [file 13007_2019_537_MOESM1_ESM.pdf]

TasselNetv2: in-field counting of wheat spikes with context-augmented local regression networks—Supplementary Materials

Haipeng Xiong[1], Zhiguo Cao[1], Hao Lu[1*], Simon Madec[2], Liang Liu[1] and Chunhua Shen[3]

1 Generation of Density Maps

We generate density maps from the images with dot annotations. At each dot, a Gaussian kernel, with a specific standard deviation, is diffused to generate the density map. The total count could be reflected by integrating over the whole density map. An example of generated density map from dotted annotations is shown in Fig. S1.

Notice that the count 593.40 is not an integer, because when wheat spikes appear at the border of the image, dots cannot diffuse beyond the image region. These parts will not be contained in the density map. Yet, this paradigm naturally takes a portion of object that near the image border into account, which strictly should not be considered as a complete object.

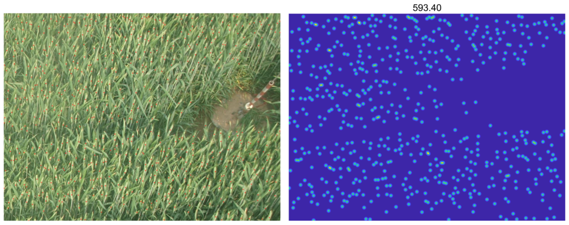

Figure S1 An example of generated density map. The left is an image with dotted annotation, and the right is the density map generated from the left image. The number of wheat spikes of the image is obtained by integrating over the density map.

2 A Brief Introduction to TasselNet

Since TasselNetv2 is built upon TasselNet [1], we give a brief introduction to TasselNet for readers to better grasp the main idea of TasselNetv2.

2.1 Overall Pipeline of TasselNet

TasselNet is a typical CNN-based local counts regression model. Fig. S2 illustrates the technical pipeline of TasselNet. First, dense sampling over the input image is executed to obtaining a large number of local image patches. These patches are then fed to TasselNet to regress the corresponding local counts individually. Next, all local counts are merged and normalized to integrate the final count map. The whole image count can be computed by summing over the count map.

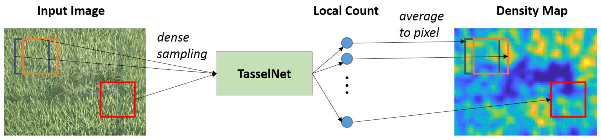

Figure S2 The processing pipeline of TasselNet. Dense sampling is first executed to collecting a large number of local patches. These patches are then fed to TasselNet successively to obtain corresponding local counts. Finally, all local counts are merged and normalized to integrate the final count map. The global image count can be acquired by summing over the count map.

TasselNet utilizes an Alex-like architecture shown in Fig. S6. ℓ_1 loss between the predicted local counts and the ground-truth local counts is adopted as the loss function to drive the learning process.

2.2 Benefits of TasselNet

In general, the success of TasselNet can be ascribed to the following reasons:

- **Sufficient training samples:** Since TasselNet processes densely-sampled local image patches rather than the whole images, a large number of training samples can be guaranteed. In the WSC dataset, images used for training are only $1,359$, but the number of sampled patches reaches $5,357,178$ when densely sampling 64×64 local patches with a stride of 16.
- **Appropriate regression target:** The loss function measures the error of local counts, instead of the error of density maps. Regressing the density map is strict, because it drives the network to achieve pixel-level accuracy. Generating accurate ground-truth density maps, however, perhaps is almost impossible for wheat spikes with only a fixed Gaussian kernel. As shown in Fig. S3, when adopting different standard deviations for the Gaussian kernel, density maps differ significantly, while local counts almost remain the same. Thus, regressing local counts is a

*Correspondence: poppinace@hust.edu.cn
[1]National Key Laboratory of Science and Technology on Multi-Spectral Information Processing, School of Artificial Intelligence and Automation, Huazhong University of Science and Technology, Wuhan, 430074, PR China
Full list of author information is available at the end of the article

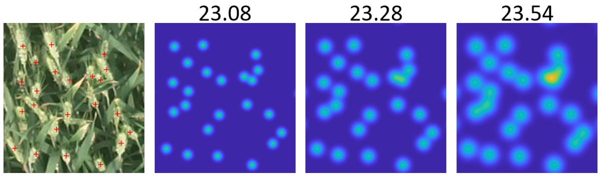

Figure S3 Density maps generated with different standard deviations of the Gaussian kernel. The leftmost image is the raw wheat image, and the remaining images are density maps generated by the Gaussian kernel with 4,6 and 8 as the standard deviation respectively. Numbers above the density maps represent the spike count, obtained by summing over the density maps.

key property that enables TasselNet for robust regression. This may also be true for other non-rigid objects, like wheat spikes.

2.3 Drawbacks of TasselNet

TasselNet also has inherit limitations:

- **Lack of contextual information:** TasselNet treats local patches independently and processes them separately, especially during the training. Since wheat spikes may partially present in local patches, the information outside the patches is invisible to TasselNet. It is thus difficult to give a true response to these patches. In these scenarios, our intuition tells us that contextual information may help, because additional information, such as the stem or the remaining part of wheat spikes, can imply clearly whether there indeed exists wheat spikes or not. It is worth noting some counting models [2, 3] unconsciously exploit the context during inference due to the fully convolutional paradigm (we will show later that fully convolutional network can implicitly make use of the context, but we remark that this point is not explicitly mentioned in existing literatures), but they do not utilize such information during training. As we will show in our experiments, this gives the problem of *information asymmetry*, which negatively affects the counting performance.
- **Waste of receptive field:** Through analyzing the receptive field of TasselNet, we can find the size of the receptive field of the output layer is 94×94, while the input size is only 64×64. This means 53.64% receptive field has no valid pixels, and this part is completely wasted.

These unfavorable factors seriously restrict the counting performance. *Is it possible to combine contextual information by expanding the effective receptive field?* We show such a feasibility with TasselNetv2.

3 Relationship Between TasselNetv2 and Existing Fully Convolutional Models

Given the simplicity of TasselNetv2, one may wonder what is the difference between TasselNetv2 and other existing fully convolutional architectures. Here we clarify that TasselNetv2 indeed has some distinctions due to the problem nature of object counting. We discuss these points from following two aspects:

- **FCNs in Semantic Segmentation:** The fully convolutional model was first introduced in [4] for semantic segmentation. In this dense prediction task, decoding is commonly used at the back end of the networks, in order to recover details [4, 5, 6, 7, 8]. However, in the context of object counting, since one only cares about object counts within local areas, decoding is unnecessary. More importantly, a counting network actually does not require a large receptive field because *counting is a local problem by nature*. This may be the reason why many small networks with weak context still work well.
- **FCNs in Object Counting:** CCNN [2], MCNN [9] and CSRNet [10] adopt a fully convolutional manner (without upsampling) to regress down-sampled local density maps. Their regression targets, however, are harsher than local counts, because ground-truth density maps fail to reach pixel-level accuracy. Count-ception [3] utilizes a FCN-like architecture to regress redundant counts, but it believes the networks can count everything within the receptive field. Albeit fully convolutional, local patches without the context are fed to the network due to the specialized receptive field. We argue that it is necessary to use a portion of the receptive field as local visual contextual information (objects in the context are not summed up into the local count) to achieve accurate counting.

4 Details of TasselNetv2

4.1 Number of Parameters

We show the parameters of TasselNet and TasselNetv2 in Table S1 layer by layer to explain why they own the same number of parameters.

4.2 Receptive Field of TasselNetv2

The receptive field denotes the region in the input image that influences each element in the output map. A detailed tutorial about the calculation of the receptive field of CNNs can be found at:

`https://medium.com/mlreview/a-guide-to-receptive-field-arithmetic-for-convolutional-neural-networks-e0f514068807`.

It can be computed following the Algorithm S1, and a tool for calculating the receptive field can be found at:

Table S1 Layer by layer comparison towards the number of parameters of TasselNetv2 and TasselNet. For convolutional layers, we compute the number of parameters as $kernel\ size \times kernel\ size \times input\ channels \times output\ channels$, and $input\ numbers \times input\ numbers$ for fully connected layers.

Layers	TasselNet	TasselNetv2
conv1	$3 \times 3 \times 3 \times 16$	$3 \times 3 \times 3 \times 16$
conv2	$3 \times 3 \times 16 \times 32$	$3 \times 3 \times 16 \times 32$
conv3	$3 \times 3 \times 32 \times 64$	$3 \times 3 \times 32 \times 64$
conv4	$3 \times 3 \times 64 \times 64$	$3 \times 3 \times 64 \times 64$
conv5	$3 \times 3 \times 64 \times 64$	$3 \times 3 \times 64 \times 64$
conv6(fc1)	$(8 \times 8 \times 64) \times 128$	$8 \times 8 \times 64 \times 128$
conv7(fc2)	128×128	$1 \times 1 \times 128 \times 128$
conv8(fc3)	128×1	$1 \times 1 \times 128 \times 1$
Total	6.38×10^5	6.38×10^5

Algorithm S1: Compute the Receptive Field

Input: Layer setting of the CNN
Output: Size and stride of the receptive field $RFsize$, $RFstride$

```
Initialize RFsize = 1, RFstride = 1;
for layer ← 1 to end do
    Get layer size and stride Lsize, Lstride;
    RFsize ← RFsize + (Lsize − 1) × Lstride;
    RFstride ← RFstrdie × Lstride;
return RFsize, RFstride
```

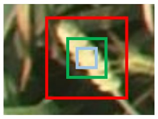

Patch Size	MAE	RMSE
16	182.96	247.44
32	64.80	101.39
64	**36.06**	**62.61**

Figure S4 Capturing a wheat spike with different patch sizes (16×16, 32×32, 64×64). Only when the local patch size reaches 64×64, it can capture the whole spike. The right table concludes the performance of TasselNet on the validation set of the WSC dataset under various sampling patch sizes.

https://fomoro.com/research/article/receptive-field-calculator.

Table S2 shows the receptive fields of TasselNetv2 layer-by-layer. Context is naturally exploited in TasselNetv2 by most local areas, but context close to image borders is partially utilized by TasselNetv2, e.g., the local area in the upper left corner only has the lower right part of the context. In order to keep the size of these local areas to 94×94, we need to fix 15 zero paddings around the borders of the input image. In TasselNetv2, this pre-processing is achieved by the accumulation of the zero paddings in the first five layers (these zero paddings accumulate to 15 zero paddings around the input image, as shown in Table S2).

4.3 Detailed Pre-trained Model Setting

Both of the TasselNet and TasselNetv2 can replace the feature extractors (the first 5 convoltional layers) with all of the convolutional layers in VGG16 [11] to further boost the counting performance. We show the network architectures of TasselNet (VGG16-pre) and TasselNetv2 (finetuned with pre-trained VGG16) layer by layer in Table S3.

5 Detailed Experiments

5.1 Searching Optimal Settings

The local patch size and the standard deviation of Gaussian kernel are two vital hyper parameters that determine the local visual patterns of spikes. Since TasselNet is the direct baseline of TasselNetv2, we set the hyper parameters of TasselNetv2 same as the TasselNet, in order to demonstrate the superiority of TasselNetv2 w.r.t. TasselNet and the benefit of embedding context information. Hence, we first search the optimal parameters on the WSC dataset using TasselNet so that TasselNet can report the optimal performance, and we then apply the same parameters to TasselNetv2.

In addition, three different network architectures are chosen as the backbone of TasselNet and are evaluated to find the best architecture on the WSC dataset.

Local Patch Size Local patches are the input of TasselNet. We evaluate three different local patch sizes ranging from 16×16 to 64×64. Results on the validation set are shown in Fig. S4. We observe that the increased patch size leads to the decreased counting error, and 64×64 performs the best. These results are consistent with our perceptual justification. The local patch size determines whether the network can "see" a complete wheat spike or not. When the local patch size is small, local patches cannot cover the whole spike, so this type of visual patterns are missing to the network. The poor counting performance is not surprising. As a consequence, it is generally encouraged to use a large enough patch size that can cover the size of the object.

It is interesting to ask: could the local patch size continue to increase? To answer this, we further analyze the number of wheat spikes within local patches on the training set. According to Table S4, when the local patch size increases, the number of local patches used for training drops rapidly, but the range of spike counts within local patches expands at the same time. This means more diverse local patterns will be fitted with less training samples, which may cause the problem of overfitting.

In addition, large local patch sizes also increase the error of merging and normalization process after dense sampling. An additional experiment is performed to justify this point. First, each ground-truth density map is densely sampled to a set of local density maps. Then, all of these density maps are summed separately to

Table S2 Receptive fields of TasselNetv2. $filter\ size$ refers to the numbers of nodes in this layer. $rf\ size$ refers to the size of receptive field. $rf\ stride$ refers to the sliding stride of receptive Field. $accumulative\ padding$ refers to the cumulative of zero paddings around the borders of the input image.

	conv1	max pool1	conv2	max pool2	conv3	conv4	conv5	max pool3	conv6	conv7	conv8
fliter size	3×3	2×2	3×3	2×2	3×3	3×3	3×3	2×2	8×8	1×1	1×1
filter padding	1	0	1	0	1	1	1	0	0	0	0
fliter stride	1	2	1	2	1	1	1	2	2	1	1
rf size	3×3	4×4	8×8	10×10	18×18	26×26	34×34	38×38	94×94	94×94	94×94
rf stride	1	2	2	4	4	4	4	8	16	16	16
accumulative padding	1	1	3	3	7	11	15	15	15	15	15

Table S3 layer by layer setting of TasselNet (VGG16-pre) and TasselNetv2 (finetuned with pre-trained VGG16). The convolutional layers are defined in the format: $kernel\ size \times kernel\ size \times input\ channels \times output\ channels,\ padding,\ /stride$, pooling layers in the format: $kernel\ size \times kernel\ size,\ padding,\ /stride$, fully connected layers in the format: $input\ numbers \times input\ numbers$.

Layers	TasselNet (VGG16-pre)	TasselNetv2 (VGG16-pre)
conv1_1	$3 \times 3 \times 3 \times 64,\ 1,\ /1$	$3 \times 3 \times 3 \times 64,\ 1,\ /1$
conv1_2	$3 \times 3 \times 64 \times 64,\ 1,\ /1$	$3 \times 3 \times 64 \times 64,\ 1,\ /1$
maxpool1	$2 \times 2,\ 0,\ /2$	$2 \times 2,\ 0,\ /2$
conv2_1	$3 \times 3 \times 64 \times 128,\ 1,\ /1$	$3 \times 3 \times 64 \times 128,\ 1,\ /1$
conv2_2	$3 \times 3 \times 128 \times 128,\ 1,\ /1$	$3 \times 3 \times 128 \times 128,\ 1,\ /1$
maxpool2	$2 \times 2,\ 0,\ /2$	$2 \times 2,\ 0,\ /2$
conv3_1	$3 \times 3 \times 128 \times 256,\ 1,\ /1$	$3 \times 3 \times 128 \times 256,\ 1,\ /1$
conv3_2	$3 \times 3 \times 256 \times 256,\ 1,\ /1$	$3 \times 3 \times 256 \times 256,\ 1,\ /1$
conv3_3	$3 \times 3 \times 256 \times 256,\ 1,\ /1$	$3 \times 3 \times 256 \times 256,\ 1,\ /1$
maxpool3	$2 \times 2,\ 0,\ /2$	$2 \times 2,\ 0,\ /2$
conv4_1	$3 \times 3 \times 256 \times 512,\ 1,\ /1$	$3 \times 3 \times 256 \times 512,\ 1,\ /1$
conv4_2	$3 \times 3 \times 512 \times 512,\ 1,\ /1$	$3 \times 3 \times 512 \times 512,\ 1,\ /1$
conv4_3	$3 \times 3 \times 512 \times 512,\ 1,\ /1$	$3 \times 3 \times 512 \times 512,\ 1,\ /1$
maxpool4	$2 \times 2,\ 0,\ /2$	$2 \times 2,\ 0,\ /2$
conv5_1	$3 \times 3 \times 512 \times 512,\ 1,\ /1$	$3 \times 3 \times 512 \times 512,\ 1,\ /1$
conv5_2	$3 \times 3 \times 512 \times 512,\ 1,\ /1$	$3 \times 3 \times 512 \times 512,\ 1,\ /1$
conv5_3	$3 \times 3 \times 512 \times 512,\ 1,\ /1$	$3 \times 3 \times 512 \times 512,\ 1,\ /1$
maxpool5	$2 \times 2,\ 0,\ /2$	$2 \times 2,\ 0,\ /2$
conv6(fc1)	$(2 \times 2 \times 512) \times 512$	$2 \times 2 \times 512 \times 512,\ 0,\ /1$
conv7(fc2)	512×512	$1 \times 1 \times 512 \times 512,\ 0,\ /1$
conv8(fc3)	512×1	$1 \times 1 \times 512 \times 1,\ 0,\ /1$
Total	1.60×10^7	1.60×10^7

Table S4 The numbers of local patches used for training TasselNet with different local patch sizes.

Patch Size	16	32	64	128
Patch num	9.20×10^7	2.24×10^7	5.36×10^6	1.19×10^6

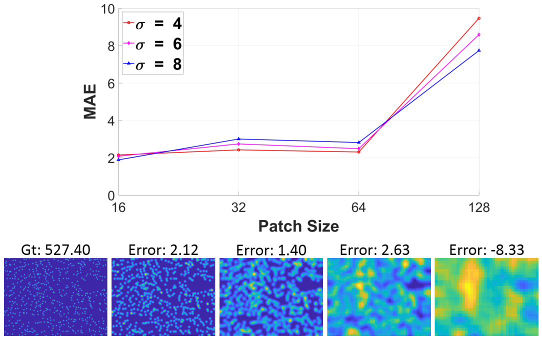

Figure S5 The error of recovering count map by merging and normalization process. The top figure shows the error curves with different patch sizes and standard deviations σ, while the bottom line presents an example of count maps generated under various patch size (16, 32, 64, 128). The leftmost image is ground truth density map, and the remaining images are generated with increasing patch sizes from left to right. The larger the dense sampling patch size, the poorer the quality of the resulting count map.

compute ground-truth local counts in the corresponding local areas. No error is introduced in these two steps. Finally, all ground truth local counts are merged and normalized to obtain the global count map. The global count map is further summed to obtain the global count. After this step, counting errors occur. The reason is that, after dense sampling, local areas have overlaps, and the merging and normalization process cannot exactly restore ground-truth density map from local count values only, as shown in Fig. S5. The larger the local patch size is, the worse the quality of the count map presents. A large local patch size, such as 128×128, will cause a notable counting error. Quantitative results are consistent with the following observations, when setting the local patch size less than 64, the errors are almost negligible. When the local patch size reaches 128×128, the error increases significantly. This suggests the local patch size should not exceed 128×128.

Table S5 The performance of TasselNet on the validation set of the WSC dataset using different Gaussian kernels. The best performance is boldfaced.

Gaussian Size	4	6	8
MAE	**36.06**	36.61	36.96
RMSE	62.61	61.09	**58.88**

As a summary of our evaluations, 64×64 is chosen as the local patch size, and it may not further increase.

Gaussian Kernel Size Gaussian smoothing is employed to generate density maps from wheat images with dotted annotations, and the Gaussian kernel size should be set appropriately to fit the sizes of most wheat spikes. Here we generate ground-truth density maps with different standard deviations of Gaussian kernel and train TasselNet respectively. Quantitative results are presented in Table S5. We observe that 4 is the optimal standard deviation of Gaussian kernel (36.06 MAE). When the standard deviation increases to 6 or 8, counting performance only slightly decreases. This suggests regressing local counts is somewhat insensitive to specific Gaussian kernel sizes. In the following experiments, the standard deviation of Gaussian kernel is fixed to 4.

Backbone of TasselNet Three network architectures similar to AlexNet [12], VGG16 [11] and ResNet18 [13] are chosen as the backbone of TasselNet. Fig. S6 shows the architectures of TasselNet with different backbones. 3×3 is adopted as the filter size of the convolutional layers, with the padding size of 1 to maintain the size of feature maps. Strides of all convolutional layers equal to 1, and only the pooling layers reduce the resolution of feature maps. After each max pooling layer, the number of feature map channels is doubled to make up the loss of information. We train each network and monitor the performance on the validation set to terminate the optimization. In particular, we focus on the generalization ability of different networks, so we directly report the counting performance on the test set in Table S6.

We found that, AlexNet-like architecture is the most suitable backbone for TasselNet, with the lowest network capacity at the meantime (MAE is 61.35 and the number of parameters is 6.38×10^5). ResNet-like architecture slightly increases the model capacity but performs slightly poor (MAE is 62.36 and the number of parameters is 7.09×10^5). But when adopting VGG16-like architecture, counting performance significantly declines, and the network capacity also dramatically increases (MAE is 66.53 and the number of parameters increases to 9.12×10^6). This strange results can be attributed to the differences in distribution between the training set and test set, as shown in Fig. S8. VGG16-like TasselNet fits the training set much better than AlexNet-like TasselNet (lower training error of VGG16-like TasselNet in Fig. S7), but it fails to generalize well on the test set due to the distribution differences.

Overall, we choose the AlexNet-like architecture as the backnone of TasselNet.

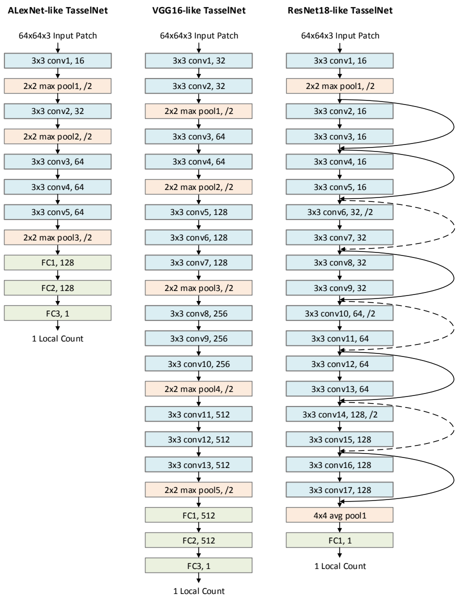

Figure S6 Three different architectures used as the backbone of TasselNet. The definitions of convolution and pooling layers are in the format: fliter size + layer name, number of channels, /stride. And fully connected layers are defined in the format: layer name, number of nodes. For ResNet18, black solid arcs indicate direct addition, while black dotted arcs indicate a 1×1 convoution to make numbers of channels the same is needed before addition.

Table S6 The performance of TasselNet [1] on the test set of the WSC dataset using various network architecture as backbone. *Parameters* denotes the number of parameters in the network. The best performance is boldfaced.

Network Architecture	MAE	RMSE	#Parameters
AlexNet-like	**61.35**	99.27	6.38×10^5
VGG16-like	66.53	107.14	9.12×10^6
ResNet18-like	62.36	**96.15**	7.09×10^5

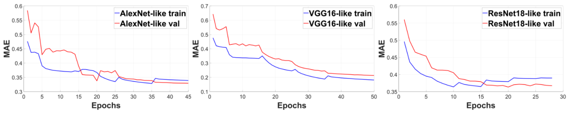

Figure S7 Training curves of AlexNet-like, VGG16-like and ResNet18-like TasselNet. Although the training error fluctuates, the validation error always decreases, which means that the network is trained smoothly.

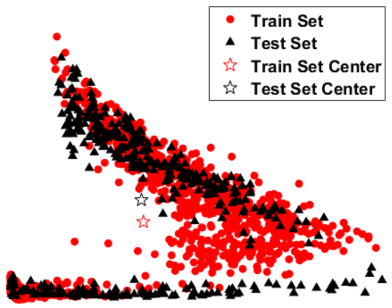

Figure S8 Data distribution visualization of the training set and test set. We first obtain the feature maps of the last convolutional layer, then acquire the average response per channel via global pooling the feature maps, and finally utilize two channels of the feature maps to visualize the data distribution. These two channels have the greatest responses to all samples, thus possible to generalize the distribution of feature space.

Table S7 The Performance of TasselNet and TasselNetv2 on the test set of the WSC dataset. All networks are trained from scratch. Training time for one epoch and the frame rate for processing 912×1216 images are reported. The best performance is boldfaced.

Method	MAE	RMSE	Train	Inference
TasselNet	61.35	99.27	$3495.29s$	$1.05fps$
TasselNetv2	50.79	**80.66**	$333.27s$	$13.82fps$

Training Strategies for TasselNetv2

TasselNetv2 could be trained from scratch or finetuned from pre-trained models. We evaluate these training schemes on TasselNetv2 to justify which scheme is more effective.

Training From Scratch TasselNet processes local patches with the context, while TasselNetv2 directly receives whole images. We train the networks from scratch and list the quantitative results in Table S7. It can be observed that TasselNet not only presents a huge improvement on counting performance, but also more than 10 times faster than TasselNet during the training and testing stages.

Finetuning TasselNetv2 with Pre-trained Models To enable TasselNetv2 to capture the local patterns smoothly, two pre-trained models are evaluated:

i) **TasselNet:** Since the sizes of convolutional kernels of TasselNetv2 are exactly the same as that of TasselNet, we can use the parameters of TasselNet for initialization, and then finetune TasselNetv2 using the full-image training scheme to utilize the contextual information.

ii) **VGG16 pretrained on the ImageNet:** The training procedure of TasselNetv2 is much more efficient than TasselNet, which enables TasselNetv2 to be finetuned with other sophisticated pre-trained models within reasonable amount of time. Notice that the convolutional layers of VGG16 (trained on the ImageNet) are always good feature extractors, we replace the feature extractors in TasselNetv2 (the first 5 convoltional layers) with all of the convolutional layers in VGG16 [11]. Due to 5 pooling layers in VGG16, we change the 6-th convolutional layers of TasselNetv2 to 2×2 to maintain the same output size. The detailed settings are listed in Table S3.

Quantitive results are displayed in Table S8, we find that TasselNetv2 can be quickly finetuned from pre-trained TasselNet and performs slightly better than it trained from scratch. A possible explanation may be that TasselNet gives a better parameter initialization to TasselNetv2 than purely training it from scratch.

Finetuning TasselNetv2 with pre-trained VGG16 further boosts counting performance, which suggests pre-trained VGG16 is a better backbone. To justify that the improvement is not mainly brought by pre-trained VGG16, We add another experiment of TasselNet with pre-trained VGG16 in Table S8), which uses the convolutional layers of pre-trained VGG16 to replace the first five layers of TasselNet. In this way, TasselNetv2 (VGG16-pre) can be viewed as the contextual extension of TasselNet (VGG16-pre). We find TasselNet can only reach MAE 56.97 with pre-trained VGG16. But after adding context in TasselNetv2 (VGG16-pre), MAE drops by 12.7. It is clear that the increment in TasselNetv2 (VGG16-pre) is mainly brought by adding the context. Meanwhile, we notice that training TasselNet (VGG16-pre) is time-consuming (12.38h for just one epoch), although TasselNet maintains the same number of parameters as TasselNetv2 (VGG16-pre). This is mainly due to the redundant calculations in the patch training scheme, and suggests patch training is not a profound way to train large CNN models.

However, it should be noted that the enhanced performance comes at a cost of much expensive computations—the number of parameters increases dramatically from 6.38×10^5 to 1.60×10^7 and both of the training and testing procedures are much slower (though still an order of magnitude faster than TasselNet (VGG16-pre)) .

Overall, we find TasselNetv2 is able to achieve good performance when trained from scratch with a tiny AlexNet-like network in Fig. S6, while finetuned from large pre-trained VGG16 can receive further improvement. In practice, one needs to trade off the performance and efficiency according to certain application scenarios and choose the suitable capacity for TasselNetv2.

Table S8 Finetuning TasselNetv2 with various pre-trained networks. The counting performance on the test set of the WSC dataset is shown in detail. The time for training one epoch and the frame rate for processing images with the resolution of 912×1216 are reported. The best performance is boldfaced.

	Pre-trained Network	MAE	RMSE	#Parameters	Train Time	Test speed
TasselNet	VGG16	56.97	97.08	1.60×10^7	12.38h	0.05fps
TasselNetv2	TasselNet	48.61	80.91	6.38×10^5	0.09h	13.82fps
TasselNetv2	VGG16	**44.27**	**67.47**	1.60×10^7	0.51h	3.44fps

6 Some Predictions of TasselNetv2

Fig. S9 and S10 shows some counting results of TasselNetv2 (finetuned with pre-trained VGG16) on the test set of the MTC dataset and the ShanghaiTech dataset, respectively. TasselNetv2 can predicts precise counts in most cases, while it still exposes under-estimate phenomena in some cases, particularly when severe overlapping and heavy blurring occur. This case is even hard for a human expert to count the objects (people or maize tassels) accurately. Efforts still should be paid to overcome these challenges.

It's worth mentioning that the ground truth counts are defined differently in the two datasets. On the MTC dataset, the ground truth count is defined as the sum of the density map. This kind of count naturally takes a portion of object that near the image border into account (as mentioned in Sec. 1) and is often not a integer. However, the ground truth count on the ShanghaiTech is denfined as the number of point annotations (a dot is marked at each object) in a image, which ignores partly objects near the border and treats these partly objects as whole objects.

Author details
[1]National Key Laboratory of Science and Technology on Multi-Spectral Information Processing, School of Artificial Intelligence and Automation, Huazhong University of Science and Technology, Wuhan, 430074, PR China. [2]INRA-EMMAH-CAPTE, 84914 Avignon, France. [3]School of Computer Science, The University of Adelaide, Adelaide, SA 5005, Australia.

References

1. Lu, H., Cao, Z., Xiao, Y., Zhuang, B., Shen, C.: TasselNet: counting maize tassels in the wild via local counts regression network. Plant Methods **13**(1), 79–95 (2017)
2. Oñoro-Rubio, D., López-Sastre, R.J.: Towards perspective-free object counting with deep learning. In: Proc. European Conference on Computer Vision (ECCV), pp. 615–629 (2016)
3. Cohen, J.P., Boucher, G., Glastonbury, C.A., Lo, H.Z., Bengio, Y.: Count-ception: Counting by fully convolutional redundant counting. In: Proc. IEEE International Conference on Computer Vision Workshop (ICCVW), pp. 18–26 (2017)
4. Long, J., Shelhamer, E., Darrell, T.: Fully convolutional networks for semantic segmentation. In: Proc. IEEE Conference on Computer Vision and Pattern Recognition (CVPR), pp. 3431–3440 (2015)
5. Badrinarayanan, V., Kendall, A., Cipolla, R.: SegNet: A deep convolutional encoder-decoder architecture for image segmentation. IEEE Transactions on Pattern Analysis and Machine Intelligence **39**, 2481–2495 (2017)
6. Lin, G., Milan, A., Shen, C., Reid, I.D.: RefineNet: Multi-path refinement networks for high-resolution semantic segmentation. In: Proc. IEEE Conference on Computer Vision and Pattern Recognition (CVPR), pp. 1925–1934 (2017)
7. Zhao, H., Shi, J., Qi, X., Wang, X., Jia, J.: Pyramid scene parsing network. In: Proc. IEEE Conference on Computer Vision and Pattern Recognition (CVPR), pp. 6230–6239 (2017)
8. Chen, L.-C., Papandreou, G., Kokkinos, I., Murphy, K., Yuille, A.L.: DeepLab: Semantic image segmentation with deep convolutional nets, atrous convolution, and fully connected crfs. IEEE Transactions on Pattern Analysis and Machine Intelligence **40**(4), 834–848 (2018)
9. Zhang, Y., Zhou, D., Chen, S., Gao, S., Ma, Y.: Single-image crowd counting via multi-column convolutional neural network. In: Proc. IEEE Conference on Computer Vision and Pattern Recognition (CVPR), pp. 589–597 (2016)
10. Li, Y., Zhang, X., Chen, D.: CSRNet: Dilated convolutional neural networks for understanding the highly congested scenes. In: Proc. IEEE Conference on Computer Vision and Pattern Recognition (CVPR), pp. 1091–1100 (2018)
11. Simonyan, K., Zisserman, A.: Very deep convolutional networks for large-scale image recognition. Computer Science (2014)
12. Krizhevsky, A., Sutskever, I., Hinton, G.E.: Imagenet classification with deep convolutional neural networks. In: Advances in Neural Information Processing Systems (NIPS), pp. 1097–1105 (2012)
13. He, K., Zhang, X., Ren, S., Sun, J.: Deep residual learning for image recognition. In: Proc. IEEE Conference on Computer Vision and Pattern Recognition (CVPR), pp. 770–778 (2015)

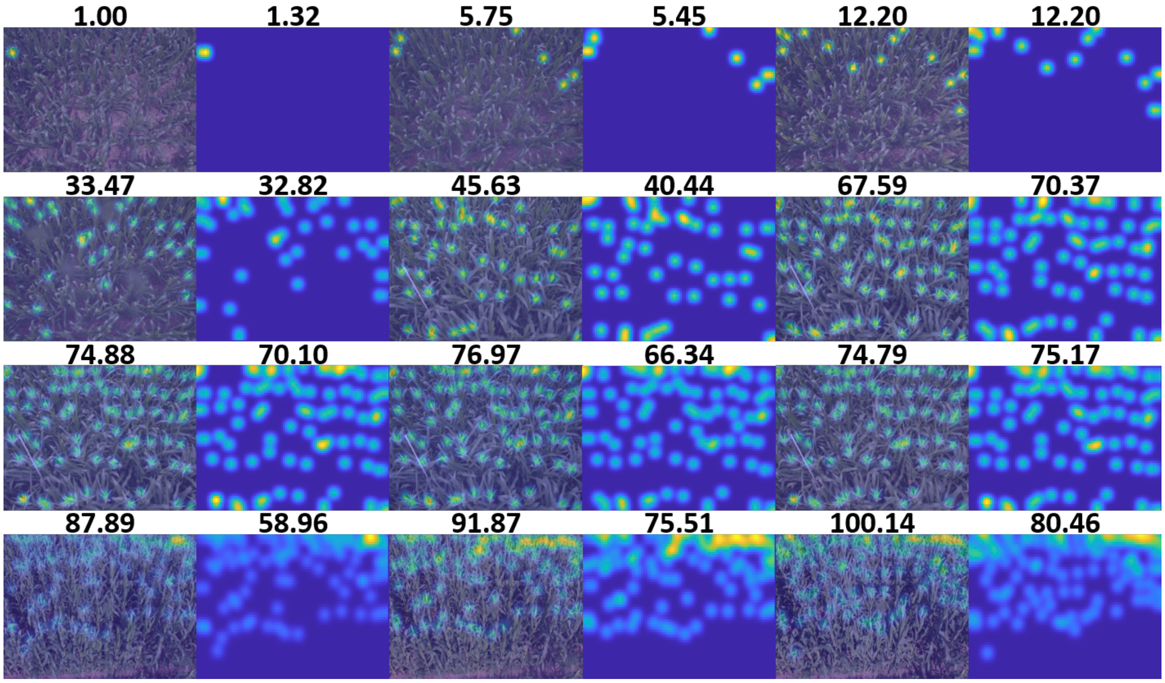

Figure S9 Some ground truth density maps overlaid on original images on the test set of the MTC dataset and count maps generated by TasselNetv2 (finetuned with pre-trained VGG16). The number above each original image denotes the ground truth count number of maize tassels, while that above each density map denotes prediction count number.

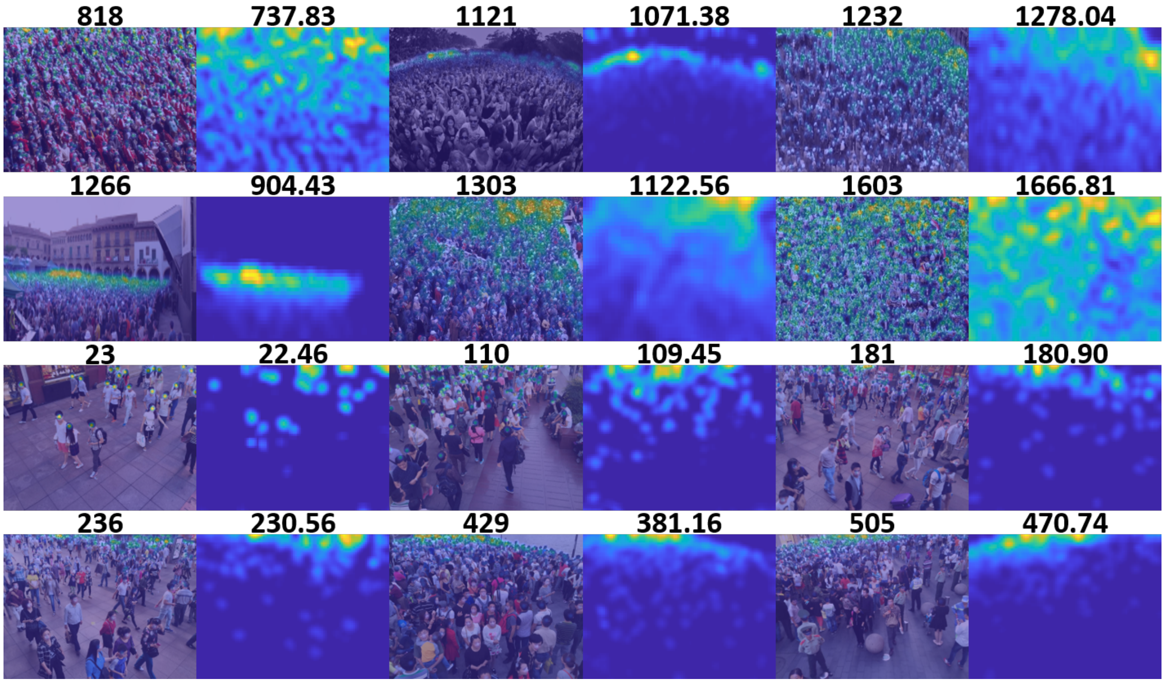

Figure S10 Some ground truth density maps overlaid on original images on the test set of the ShanghaiTech dataset and count maps generated by TasselNetv2 (finetuned with pre-trained VGG16). The number above each original image denotes the ground truth count number of people, while that above each density map denotes prediction count number. The fist two lines shows images from part A, while the last two lines displays images from part B.
